# Mapping the recognition pathway of cyclobutane pyrimidine dimer in DNA by Rad4/XPC

**DOI:** 10.1093/nar/gkad730

**Published:** 2023-09-27

**Authors:** Nikhil Jakhar, Akshay Prabhakant, Marimuthu Krishnan

**Affiliations:** Center for Computational Natural Sciences and Bioinformatics (CCNSB), International Institute of Information Technology, Gachibowli, Hyderabad 500032, Telangana, India; Center for Computational Natural Sciences and Bioinformatics (CCNSB), International Institute of Information Technology, Gachibowli, Hyderabad 500032, Telangana, India; Center for Computational Natural Sciences and Bioinformatics (CCNSB), International Institute of Information Technology, Gachibowli, Hyderabad 500032, Telangana, India

## Abstract

UV radiation-induced DNA damages have adverse effects on genome integrity and cellular function. The most prevalent UV-induced DNA lesion is the cyclobutane pyrimidine dimer (CPD), which can cause skin disorders and cancers in humans. Rad4/XPC is a damage sensing protein that recognizes and repairs CPD lesions with high fidelity. However, the molecular mechanism of how Rad4/XPC interrogates CPD lesions remains elusive. Emerging viewpoints indicate that the association of Rad4/XPC with DNA, the insertion of a lesion-sensing β-hairpin of Rad4/XPC into the lesion site and the flipping of CPD’s partner bases (5′-dA and 3′-dA) are essential for damage recognition. Characterizing these slow events is challenging due to their infrequent occurrence on molecular time scales. Herein, we have used enhanced sampling and molecular dynamics simulations to investigate the mechanism and energetics of lesion recognition by Rad4/XPC, considering multiple plausible pathways between the crystal structure of the Rad4–DNA complex and nine intermediate states. Our results shed light on the most likely sequence of events, their potential coupling and energetics. Upon association, Rad4 and DNA form an encounter complex in which CPD and its partner bases remain in the duplex and the BHD3 β-hairpin is yet to be inserted into the lesion site. Subsequently, sequential base flipping occurs, with the flipping of the 5′-dA base preceding that of the 3′-dA base, followed by the insertion of the BHD3 β-hairpin into the lesion site. The results presented here have significant implications for understanding the molecular basis of UV-related skin disorders and cancers and for paving the way for novel therapeutic strategies.

## Graphical Abstract

**Graphical Abstract:**
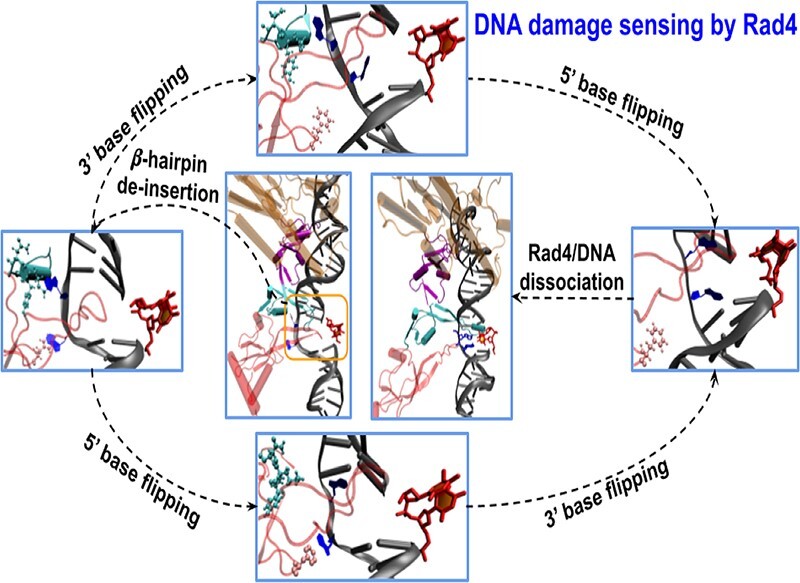

## INTRODUCTION

DNA plays a central role in the life of a biological cell. DNA damage can give rise to deleterious cellular responses and cellular malfunction, which can lead to eventual cell death or uncontrolled cell growth (1–6). DNA repair proteins can sense and repair DNA damage to safeguard the genome integrity of cells. The UV light-induced cyclobutane pyrimidine dimer (CPD) is the most prevalent DNA lesion, which is implicated in a variety of genetic skin-related diseases and cancers in humans (7–10).

The nucleotide excision repair (NER) is a key CPD repair mechanism in which the structural distortions in a CPD-containing DNA are sensed by a specific repair protein followed by the recruitment of other proteins to repair the DNA damage (11–19). A corkscrew-like sliding of the repair protein on the DNA to scan and search for the lesion, its stalling and subsequential conformational changes at the lesion site appear to be necessary for damage verification and further NER actions (20–25). The DNA damage-binding proteins (DDB1 and DDB2) are essential for the early identification of UV-induced damages in living organisms and promotes effective damage recognition by XPC (26,27). Once the damage site is identified by XPC, the transcription factor II H (TFIIH) is called upon, whose ATP-dependent helicases (XPB and XPD) unwind the duplex to open a bubble in the DNA around the lesion (28–40). Subsequently, the lesion-containing oligonucleotide is excised by endonucleases (XPG and XPF) and the resultant gap is filled by the DNA polymerase and then sealed by a DNA ligase to regenerate the intact DNA structure (41–46).

Radiation sensitive 4 (Rad4), which is a yeast orthologue of XPC with significant structural and functional similarities, continues to serve as a useful model to examine XPC-mediated DNA damage repair in humans (47–49). The availability of the crystal structure of Rad4 complexed with a CPD-containing DNA fragment permits molecular-level investigation of Rad4-mediated DNA damage repair in yeast cells, which then serves as a baseline model for XPC (47). Rad4 consists of an N-terminal transglutaminase domain (TGD), and three β-hairpin domains (BHD1, BHD2 and BHD3) (47,50). Among them, TGD and BHD1 domains bind to the undamaged segment of DNA and help maintain the structural integrity of the undamaged portion of DNA. The β-hairpin of BHD2 engages with the DNA minor groove around the lesion and forms hydrogen bonds with the DNA backbone, while the β-hairpin of BHD3 inserts into the DNA major groove to fill the gap created by the flipped-out CPD and its partner bases from the undamaged DNA strand (51,52). These expelled partner bases are held at the BHD2–BHD3 binding interface.

The presence of CPD alters the base-base hydrogen bonding patterns and affects the stability of the base pairing of the DNA in the vicinity of the damage site. Consequently, the overall structure of the DNA is distorted, leading to the bending and unwinding of the DNA at the lesion site. For instance, the CPD-containing DNA in the absence of Rad4 is bent toward the major groove by ≈30° and unwound by ≈9° (53–55) near the lesion site. These local structural distortions in DNA, which are caused by the inability of CPD to form hydrogen bonds with its partner bases (5), serve as a signal for damage recognition by repair proteins (5,10,56–58). When Rad4 binds to the damaged DNA, the bending angle of DNA changes to around 42° with significant helical unwinding near the active site (47).

Although the aforementioned structural insights derived from the Rad4–DNA crystal structure offer a static view of damage recognition by Rad4, the overall mechanism is likely to be highly dynamic in nature. Understandably, the structure-based static view cannot easily account for the complexity of various dynamic events that occur during damage recognition and repair by Rad4. For instance, previous studies have identified the association of Rad4 and DNA, flipping out of the partner bases and the lesion from the DNA duplex, and the insertion of β-hairpins into the DNA grooves as the key events of the early phase of lesion recognition by Rad4. Each of these events has specific mechanism and time scales associated with it. For example, the Rad4–DNA association is likely to involve the initial binding of TGD and BHD1 to the undamaged part of DNA, followed by the binding of BHD2 and BHD3 to the lesion-containing part. Subsequently, the BHD3 β-hairpin and BHD2 β-hairpin are expected to insert into the major and minor grooves of the DNA, respectively, causing the CPD lesion and its partner thymines to entirely flip out of the DNA duplex. Given that the partner bases could not flip out of the CPD-containing DNA duplex in the absence of Rad4 (53), it appears that the association of Rad4 with DNA must have preceded the flipping of the partner bases. However, the order of flipping of 5′ and 3′ partner bases and whether or not these flipping events succeed the insertion of the BHD3 β-hairpin into the lesion site remain obscure. The other challenge is that these molecular events are likely to be cooperative and correlated with one another. That is, a hindrance of any of these events can lead to the failure of the NER process. Thus, it is essential to elucidate the precise order of these events, their molecular mechanisms, energetics and interdependence at the atomistic level. The present work explores the mechanisms, energetics and order of these events and any possible couplings between them using molecular dynamics and enhanced sampling simulations.

## MATERIALS AND METHODS

### Models

#### Rad4–DNA complex

**Figure 1.**
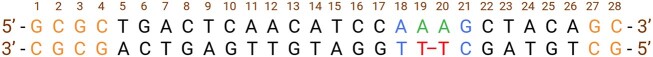
DNA sequence and nucleotide numbering scheme used in the study. The CPD (red), the partner bases (green) and the neighbouring base pairs (blue) are shown.

**Figure 2.**
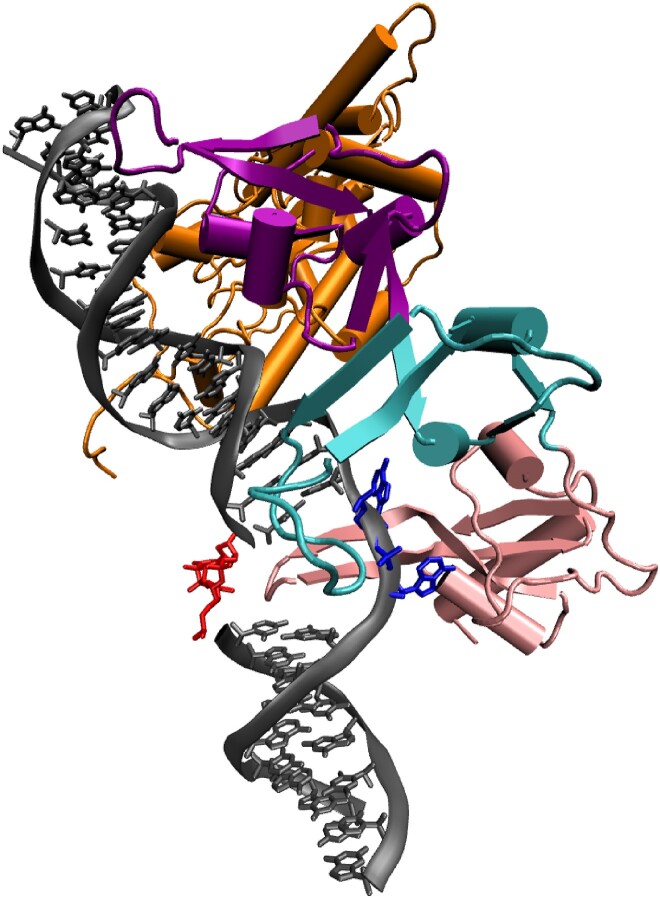
Model of the CPD-containing DNA–Rad4 complex. Here, TGD (orange), BHD1 (purple), BHD2 (cyan) and BHD3 (pink) domains of RAD4 and the CPD (red) and its partner adenine bases (blue) of DNA (grey) are shown. The image was generated using VMD (59).

The crystal structure of the Rad4–DNA complex (PDB ID: 2QSG) was used as a model of the final associated open complex (47). In this bound state, BHD2 and BHD3 of Rad4 are proximal to the damage site, the β-hairpins of BHD2 and BHD3 are inserted into the minor and major grooves, respectively, of DNA near the lesion and the CPD and its consecutive adenine partner bases on the undamaged strand are completely expelled from the DNA duplex. The DNA sequence taken from this crystal structure was extended to a 28-base pair sequence (Figure 1) in which a CPD-lesion is present at the 19th and 20th base pairs. In what follows, the CPD’s partner bases A19_*u*_, A20_*u*_ on the undamaged strand (denoted by a subscript *u*) will be referred to as 5′-dA and 3′-dA, respectively. This nucleotide sequence corresponds to a perfectly matched CPD-containing DNA, which is different from our previous studies that focused on mismatched DNA (60). Since the coordinates of the CPD lesion were not resolved in the crystal structure of the open complex, a CPD lesion was added to the open complex. In addition, each of the two mismatched thymine partner bases opposite the lesion in the crystal structure of the open complex was replaced by an adenine base using the Swapna module of UCSF Chimera (61,62). Although CPD-containing ’perfectly-matched’ DNA may be a less efficient substrate for the direct recognition by Rad4/XPC when compared to CPD within a three-base mismatch (63,64), we chose this model to investigate the Rad4–DNA binding process in the absence of mismatches, thereby probing the sole contributions of the lesion in this process. The model of the resultant associated open complex of Rad4 and DNA is shown in Figure 2.

#### Intermediates of Rad4–DNA complex

The structural models of the key intermediate states (models B, C, D, E and F in Figure 4) of the CPD-containing DNA–Rad4 complex were considered to probe the intervening processes that underlie the damage recognition and repair by Rad4/XPC. Model B corresponds to an intermediate state in which the BHD3 β-hairpin is deinserted from the final bound state of the Rad4–DNA complex. Models C and D are the same as model B, but one of CPD’s partner bases (5′-dA or 3′-dA) is flipped into the DNA duplex. Model E is also the same as Model B, but both the partner bases are flipped into the DNA duplex. In models C–E, CPD remains outside of the DNA duplex. Model F is the same as Model E, but the CPD is also flipped into the DNA duplex. Each of these models represents an end state of a plausible intervening process in the overall NER process. Here, an intervening process of interest corresponds to a transition between two such models. For instance, transitions between models A and B correspond to the deinsertion (model A → model B) or insertion (model B → model A) of the BHD3 β-hairpin from the DNA duplex. The overall NER process is thought of as a sequence of such intervening transitions between model A and model G.

**Figure 3.**
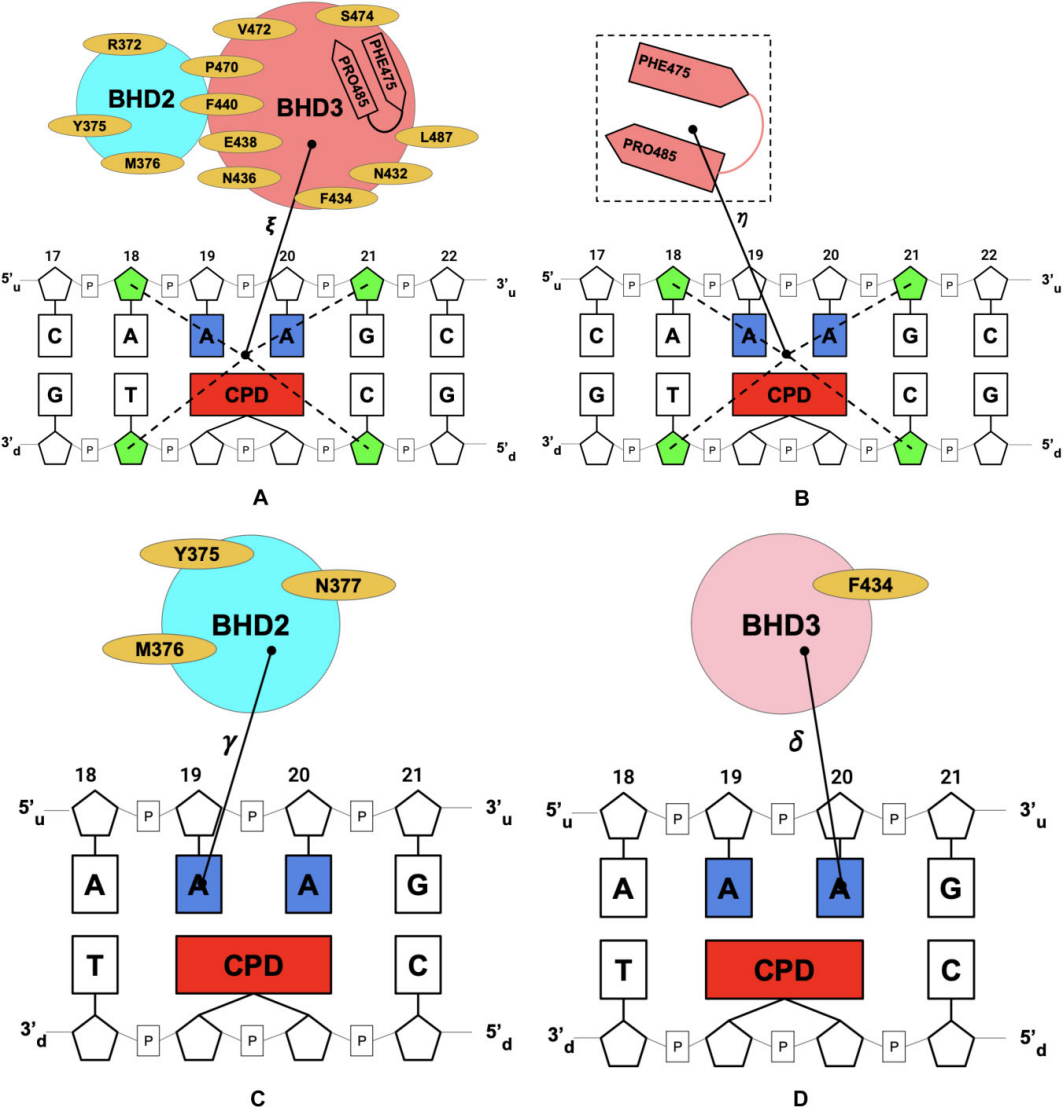
Schematic representation of the collective variables used to simulate the three key processes of NER. (**A**) ξ is the distance between the center of mass (COM) of heavy atoms of amino acids (yellow ellipses) and the backbone heavy atoms of BHD3 β-hairpin amino acids (PHE475-PRO485) (coral), and the COM of the sugar rings of the neighbouring bases of CPD and their partners (A18_*u*_, G21_*u*_, T18_*d*_, C21_*d*_) (green) (**B**) η is the distance between COM of backbone heavy atoms of the BHD3 β-hairpin (coral) and the COM of the sugar rings of the neighbouring bases of CPD and their partners (A18_*u*_, G21_*u*_, T18_*d*_, C21_*d*_) (green). (**C**) γ is the distance between COM of heavy atoms of the BHD2 domain pocket (yellow ellipses) and the adenine base of nucleotide A19_*u*_ (blue). (**D**) δ is the distance between COM of heavy atoms of PHE434 belonging to the BHD3 domain (yellow ellipse) and the adenine base of A20_*u*_ (blue).

**Figure 4.**
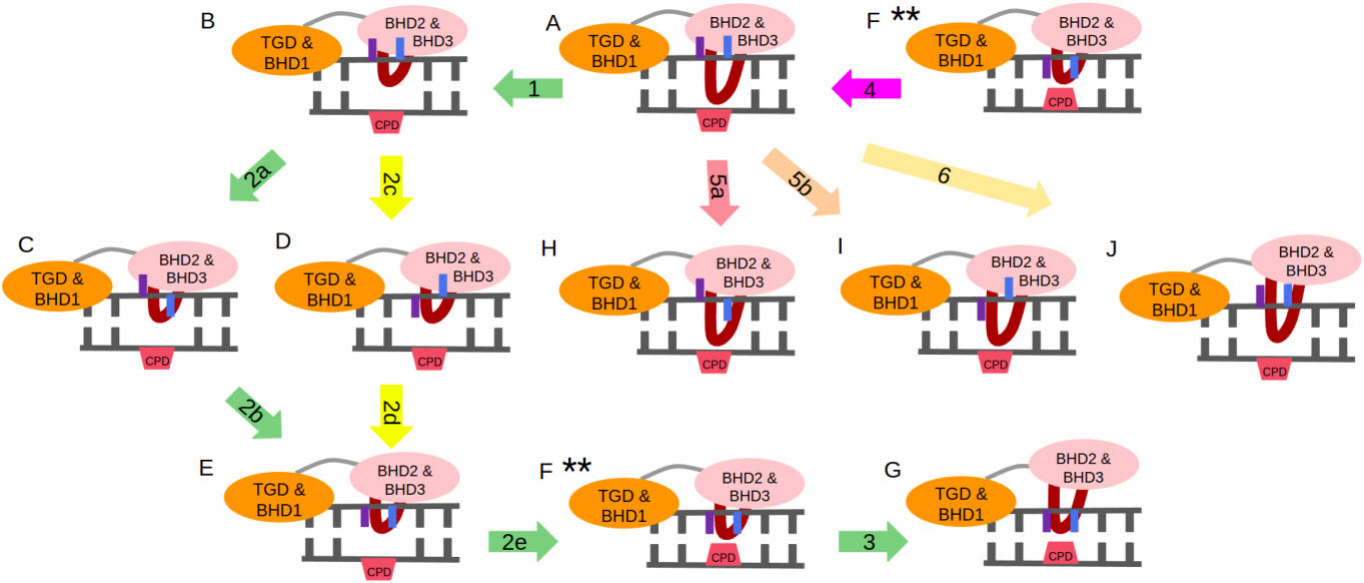
Models and sequences of events (denoted by numbered arrows) considered. (**A**) Rad4–DNA bound complex, (**B**) bound complex with BHD3 β-hairpin deinserted from the damage site, (**C**, **D**) same as (B) except for one of the partner bases (3′-dA (blue) or 5′-dA (violet)) flipped into the DNA duplex, (**E**) same as (B) but both partner bases are flipped into the DNA duplex, (F**) same as (B) except that both partner bases and the CPD lesion are flipped into the DNA duplex, (**G**) same as (E) but the BHD2 and BHD3 domains are dissociated from the DNA, (**H, I**) bound complex with one of the partner bases flipped into the DNA duplex, (**J**) bound complex with the BHD2 and BHD3 domains dissociated from the DNA. Transitions studied: deinsertion of BHD3 β-hairpin (1); flipping of partner bases (2a, 2b, 2c, 2d) followed by the flipping of CPD (2e) in the β-hairpin deinserted state; Structure marked with * was selected by taking the most probable structure after clustering on 100 ns of the unbiased production run of the bound Rad4–DNA complex. Structures marked with ** are the same meta-stable state formed after flipping in CPD.

By analyzing different intervening processes between intermediate states using appropriate collective variables (see later), we investigated various possible mechanisms for the NER process. To model these intermediate states, multiple unbiased NPT molecular dynamics (MD) simulations were carried out starting from structures with varying values of the collective variable (CV) specific to each intervening process. The CV time series from these simulations were analyzed, and a significant number of trajectories were found to converge around a specific value of the CV. The structures obtained from these trajectories with the CV value close to this converged value were then clustered using the root mean square deviation (RMSD) method with respect to key nucleotides surrounding the damaged DNA lesion site (C17_*u*_–C22_*u*_ and G17_*d*_–G22_*d*_). The metastable state for each process was then determined by selecting the center of the top-ranked cluster. A few important metastable states of the Rad4–DNA complex obtained through this method are illustrated in Figure 4.

### Molecular dynamics simulation

All-atom molecular dynamics simulations of the model systems were carried out using AMBER 2018.10 (patched with PLUMED 2.5) simulation package (65–68) with the ff14SB (69) force fields for the protein, ParmBSC1 (70) force fields for DNA, and the TIP3P (71) model for water molecules. The partial charges of atoms in the CPD lesion were assigned using the Antechamber module of AmberTools19 and the remaining force field parameters of the lesion were adopted from the general Amber force field (GAFF) (72). Each of these model complexes was solvated in a TIP3P water box with 20 Å of water padding in all directions, and 24 Na^+^ ions were added to neutralize the system. The lengths of all the bonds involving hydrogen atoms were constrained using the SHAKE algorithm (73). The three-dimensional periodic boundary conditions were employed. The long-range electrostatic interactions were treated with the particle mesh Ewald (PME) (74) approach with a direct space cut-off of 10 Å and a tolerance of 10^−5^ and with 4th order B-spline interpolation and an Ewald coefficient of 0.27511. A distance cutoff of 10 Å was also used for the long range van der Waals interactions.

In order to maintain the overall structural integrity of the DNA-Rad4 complex during the initial phase of energy minimization, strong harmonic constraints (spring constant of 100 kcal mol^−1^ Å^−2^) were applied to the crystallographically resolved atoms of the complex to hold them near their experimentally resolved positions, while weak harmonic constraints (spring constant of 10 kcal mol^−1^ Å^−2^) were applied to the unresolved atoms of the complex whose coordinates were guessed. In the next stage of energy minimization, the harmonic constraints on the unresolved atoms were removed, while those on the resolved atoms were retained. The water molecules and counter ions were unconstrained at all stages of energy minimization. Subsequently, by retaining the harmonic constraints on the resolved atoms, the energy-minimized configurations were equilibrated for 0.02 ns in the NVT ensemble at 300 K followed by 2 ns simulations in the NPT ensemble at 300 K and 1 bar. Thereafter, all the constraints were removed and the whole of each system was energy minimized and equilibrated in the NVT ensemble for 0.02 ns and then in the NPT ensemble for 2 ns. Each energy minimization run consisted of 20 000 steepest descent steps followed by 20 000 conjugate gradient steps with a convergence tolerance of 10^−4^ kcal mol^−1^ Å^−1^ (65). The pressure was maintained at 1 bar using a Berendsen barostat (75) with a pressure relaxation time of 1 ps, while the temperature was maintained at 300 K using a Langevin thermostat (76) with a collision frequency of 1 ps^−1^. The velocity Verlet (76) algorithm was used to integrate the equations of motion with a time step of 2 fs.

### Umbrella sampling

Given that the timescales of key molecular events of NER are likely to be longer than the accessible timescales of conventional MD simulations, we employed the umbrella sampling method to capture relevant conformational changes in Rad4–DNA complex that occur during NER and to quantify the associated energetics. In the following sections, we define the collective variables used in the present study for β-hairpin insertion, the flipping of the partner bases, and Rad4–DNA association.

#### Collective variable for β-hairpin insertion

To examine the insertion of the β-hairpin of the BHD3 into the lesion-site, a distance-based CV, η, was chosen. η is the distance between the center of mass (COM) of the backbone heavy atoms of all the residues in the β-hairpin of BHD3 and the COM of the sugar rings of the CPD’s neighbouring bases and their partners (A18_*u*_, G21_*u*_, T18_*d*_, C21_*d*_) (Figure 3B). The biasing harmonic force constants for the equilibration and production runs were set to 75 kcal mol^−1^ Å^−2^ and 5 kcal mol^−1^ Å^−2^, respectively. η was varied from 1 Å to 22.5 Å in steps of 0.5 Å for the umbrella sampling, corresponding to a total of 44 windows. The value of η calculated for the experimental crystal structure of the Rad4–DNA complex (model A) is 4.95 Å.

#### Collective variable for base flipping

To capture the flipping dynamics of CPD’s partner bases 3′-dA and 5′-dA of the undamaged strand of the DNA, a distance-based CV was defined separately for each partner base. As per the crystal structure of the Rad4–DNA complex, both the partner bases (3′-dA and 5′-dA) are expelled from the DNA duplex and are tightly bound to the binding pocket located at the interface between BHD2 and BHD3 domains of Rad4. In this expelled extrahelical state, 5′-dA establishes favourable interactions with a few key binding pocket residues (TYR375, MET376 and ASN377) of Rad4. However, these interactions are absent in the intrahelical state, where 5′-dA is aromatically stacked with its neighbouring A18_*u*_ base of the DNA. Hence, the distance between the centre of mass (COM) of 5′-dA and that of the heavy atoms of the binding pocket residues is considered as an appropriate CV to describe the flipping dynamics of 5′-dA. Henceforth, this CV will be referred to as γ  as shown in Figure 3C. γ  was varied from 4.0 to 19.5 Å in steps of 0.5 Å, corresponding to a total of 32 windows and the biasing harmonic force constants for the equilibration and production runs were set to 100 and 10 kcal mol^−1^ Å^−2^, respectively.

Similarly, 3′-dA aromatically stacks with PHE434 of the BHD3 domain of Rad4 in the extrahelical state, whereas this stacking interaction was absent in its intrahelical state, where it stacks with the neighbouring G21_*u*_ base. Hence, the distance between the COM of 3′-dA and the COM of PHE434 was chosen as an appropriate CV to describe the flipping dynamics of 3′-dA in the present study. Hereafter, this CV will be referred to as δ  as shown in Figure 3D. δ  was varied from 2.0 to 16.5 Å in steps of 0.5 Å, corresponding to a total of 30 windows and the biasing harmonic force constants for the equilibration and production runs were set to 100 and 10 kcal mol^−1^ Å^−2^, respectively.

#### Collective variable for Rad4–DNA association

To characterize the association of the BHD2 and BHD3 domains of Rad4 with the damaged DNA, the distance, ξ , between the COM of the sugar rings of the four neighbouring nucleotides to the lesion site (see Figure 1) and the COM of the backbone heavy atoms of the binding pocket residues 372, 375, 376, 432, 434, 436, 438, 440, 470, 472, 474-487 of BHD2 and BHD3 (refer Figure 3A) of Rad4 was chosen as the relevant CV. ξ  was varied from 10 to 30 Å in steps of 0.5 Å, corresponding to a total of 41 windows and the biasing harmonic force constants for the equilibration and production runs were set to 100 and 10 kcal mol^−1^ Å^−2^, respectively.

In the dissociated state, where Rad4 and DNA are separated from each other, it is uncertain if the CPD and its partner bases remain in their extrahelical states. On the contrary, they are extruded fully from the DNA duplex while in the bound state. Thus, we examined the dissociation of Rad4 from DNA in two different models of the partner bases, namely the intra-helical (Model F) and extra-helical (Model A) models. As mentioned previously, in Model F, the BHD3 β-hairpin is removed from the DNA duplex, while in Model A, it remains inserted. In Model F, Rad4 is expected to bind to DNA (particularly the insertion of BHD3 β-hairpin) and cause the flipping of partner bases and CPD out of the DNA duplex. However, due to the infrequency of this event, it cannot be observed within the duration of our simulations. To address this, Model A was used to examine the dissociation process after the partner bases and CPD had already flipped out from the DNA duplex.

#### Umbrella sampling protocol

The umbrella sampling simulations were carried out independently for each of the aforementioned events. The CV trajectories obtained from the umbrella sampling simulations were used to calculate the potentials of mean force (PMFs) using the weighted histogram analysis method (WHAM). The starting structure for these simulations was taken from the last frame of unbiased MD simulations. The first step of each umbrella sampling run was to displace the system to the chosen window using a harmonic biasing potential with a high spring constant (*k*_*eq*_) such that the respective CV is brought to the center of the specific window. For this purpose, a biased NPT equilibration run was carried out for 200 ps for each window. Subsequently, this was followed by 6 ns of production run at 300 K and 1 atm pressure in the NPT ensemble in the presence of a harmonic biasing potential of a weaker spring constant (*k*_*prod*_), which is considerably smaller than *k*_*eq*_. The chosen values of *k*_*eq*_ and *k*_*prod*_ are different for different molecular events of interest. These umbrella sampling simulations were carried out using the same conditions as those of unbiased MD runs, with an additional restraint on the following distances: (1) distance between the COMs of bases A18_*u*_ and T18_*d*_ restrained at 6.06 Å using a harmonic bias of 25 kcal mol^−1^ Å^−2^, (2) distance between the COMs of bases G21_*u*_ and C21_*d*_ restrained at 5.85 Å using a bias of 25 kcal mol^−1^ Å^−2^. These are the neighbouring bases of the CPD lesion and its partner adenines. As the neighbouring bases are expected to be in their respective intra-helical states during the entire course of the NER process, we constrained them in their respective crystalline state conformations in all our umbrella sampling simulations (47).

#### Order of events

The order of the aforementioned events and any potential correlation between them were studied through sequential umbrella sampling simulations. In this approach, after completing the umbrella sampling for an event, a meta-stable state structure was created from the trajectories generated, which was then used for the umbrella sampling simulation of the next event in the sequence.

Previous research on the recognition and repair of UV lesions in DNA has suggested a potential sequence of events, in which the association of BHD2/3 domains with DNA is followed by flipping out of partner bases, and then by the insertion of the BHD3 β-hairpin into DNA (77,78). However, to study the energetics of these events in this specific order, a biased simulation of Rad4–DNA association followed by flipping of partner bases and insertion of the β-hairpin of BHD3 into DNA is required. Unfortunately, the crystal structure of the Rad4–DNA complex is only available in the post-recognition state, where the aforementioned events have already happened, thus making it difficult to model the sequence of events starting from the pre-associated state. Additionally, the complex energy surface of this system with multiple pathways of varying numbers of intermediate states separated by barriers of differing heights between the pre-associated state and the post-associated bound complex further complicates the elucidation of the mechanism of the entire process. To address these challenges, we have opted to investigate the process in reverse order, starting from the experimental crystal structure of the bound Rad4–DNA complex and examining the BHD3 β-hairpin deinsertion, followed by flipping in of the partner bases and Rad4–DNA dissociation.

To examine the sequence of events involving the insertion of the β-hairpin into the DNA lesion site and the flipping of partner bases, we investigated two scenarios. Firstly, we studied the dynamics of partner base flipping before and after β-hairpin insertion. Secondly, we examined β-hairpin insertion before and after the flipping of partner bases. In the former approach, the β-hairpin may prevent the partner bases from adopting an intrahelical state once inserted, whereas in the deinserted state, the partner bases can flip in and out of the lesion site during the simulation. Similarly, in the latter approach, when the partner bases are flipped out, the β-hairpin can insert itself, but in the intrahelical state of the partner bases, the inserted state is unstable.

To fully comprehend the flipping behavior of the partner bases, it is essential to determine whether 3′-dA and 5′-dA flip out simultaneously (in a concerted manner) or one after the other (in a sequential manner) following the association of Rad4–DNA. Prior research has indicated that the sequential flipping of bases is more energetically favorable than the concerted mechanism (47,60,79). However, there is still much to uncover regarding whether the flipping of 3′-dA occurs before or after 5′-dA, as well as the corresponding energetics of these flipping events. In order to determine the sequence of flipping events of the partner bases, multiple umbrella sampling simulations were carried out using different possible flipping mechanisms. The first investigation focused on the flipping dynamics of 5′-dA in two different structures: one where 3′-dA was extrahelical (Model C shown in Figure 4), and the other where 3′-dA was intrahelical (Model D). The former model represents the flipping of 5′-dA after 3′-dA is flipped out, while the latter model demonstrates the flipping of 5′-dA prior to the flipping of 3′-dA. These flipping experiments were carried out on the metastable structure of the Rad4–DNA complex, where the BHD2 β-hairpin was partially deinserted (Figure 4), and a comparison of the flipping energy profiles from both experiments can provide insight into the flipping sequence of the partner bases during DNA damage recognition by Rad4. Additionally, the flipping of these bases from their extrahelical positions was analyzed separately in the crystal structure of the Rad4–DNA complex where the BHD3 β-hairpin is inserted perfectly into the DNA duplex (Models H and I).

In order to determine whether the insertion of the β-hairpin occurs before or after the flipping of the partner bases, umbrella sampling simulations were performed on the insertion of the BHD3 β-hairpin into a DNA duplex using two different structures of the Rad4–DNA complex. One structure had extrahelical partner bases and CPD (shown in Figure 4), while the other structure had intrahelical partner bases and CPD (also shown in Figure 4). The flipping of the CPD was induced using a harmonic biasing potential on the distance between the center of mass (COM) of the CPD and the COM of the partner bases. These experiments could provide insight into the extent of coupling between the insertion of the β-hairpin and the flipping of the partner bases.

## RESULTS AND DISCUSSION

This section presents the results of umbrella sampling simulations performed on various molecular events that take place during RAD4-induced recognition, such as BHD3 β-hairpin insertion, CPD partner base flipping and DNA–Rad4 association. It is important to note that these simulations were initiated from the crystal structure of the bound complex, and therefore the results presented here reveal the reverse pathway of this recognition process. Specifically, the simulations showcase the dissociation of Rad4 from DNA, the deinsertion of the BHD3 β-hairpin from the DNA duplex, and the flipping of the CPD and its partner bases from extrahelical to intrahelical states.

### BHD3 β-hairpin deinsertion

**Figure 5.**
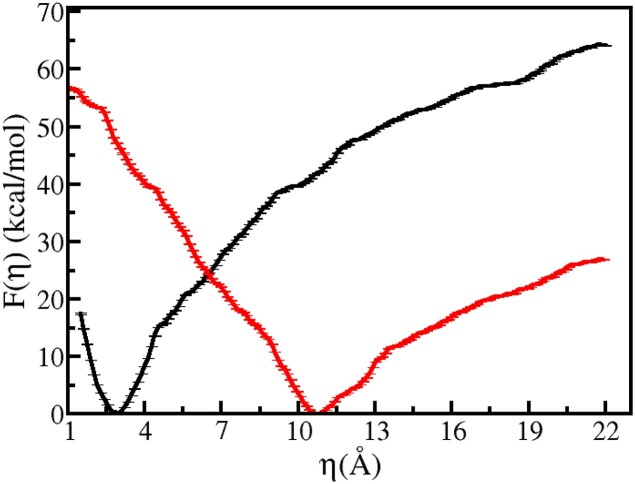
The potential of mean force for the deinsertion of the β-hairpin of BHD3 from the lesion site of the damaged DNA duplex for Model A (black) and Model F (red).

In Figure 5, the free energy profile, F(η), obtained from the umbrella sampling simulation for the deinsertion of the BHD3 β-hairpin from the Rad4–DNA complex crystal structure (Model A) is shown. The profile shows a single minimum at around η ∼ 3 Å, referred to as η_*m*_*_in_*. In this energy minimum state, the BHD3 β-hairpin stabilizes itself within the Watson–Crick double helix by establishing favourable van der Waals contacts with adjacent base pairs, including 18^th^ (A–T) and 21^st^ (G–C) base pairs (refer Figure 1), as well as partner bases. The two flipped-out partner nucleotides interact with BHD3 and form hydrogen bonds with nearby residues. These interactions help to stabilize the hairpin in the inserted state (47).

The energy minimum position is shifted by 2 Å when compared to the crystal structure of the CPD-containing mismatched DNA bound with Rad4 (47). This discrepancy can be ascribed to the dissimilarity in DNA models considered in the present study and that in the mismatched crystal structure: our model consists of a damaged but perfectly matched DNA while that in the crystal structure contains a mismatched DNA. Additionally, the CPD remains unresolved in the mismatched crystal structure. The energy basin around the minimum is a symmetric parabolic well, indicating that for small displacements around the energy minimum (i.e. Δη = ± 1.5 Å) the β-hairpin experiences an elastic restoring force due to its favourable interactions with DNA. However, for η > 4.5 Å, a noticeable deviation from this harmonic behaviour is observed. The β-hairpin breaks open its elastic cage at the lesion site by disrupting some favourable interactions and moves away from the damage site beyond this harmonic limit. This transition from the harmonic regime to the cage-breaking event is reflected in a slope change in *F*(η) at η ∼ 4.5 Å. The energy required to dislodge the β-hairpin from the lesion site of DNA in the Rad4–DNA complex is the free energy difference between the global minimum and the crossover point at η ∼ 4.5 Å, which is approximately 13 kcal mol^−1^. This energy represents the stabilization energy experienced by the BHD3 β-hairpin at the lesion site.

The analysis of umbrella sampling trajectories of the BHD3 β-hairpin’s disengagement from the DNA duplex can help identify a plausible meta-stable state of the system that occurs immediately prior to β-hairpin insertion. However, since the experimental structure of this meta-stable state is unavailable, we need to model it for two key reasons. Firstly, by starting from this state, we can investigate the mechanism and energetics of the β-hairpin insertion into the DNA duplex. Secondly, modelling this state allows us to study the independent flipping of partner bases and the β-hairpin insertion without any mutual impact on each other. To identify this deinserted metastable state, we selected specific shoulder points (8 Å ≤ η ≤ 13.5 Å) from the PMF curve where the interactions between the BHD3 β-hairpin and DNA are relatively weaker compared to those in the inserted state. Subsequently, we performed unbiased NPT MD runs (each of 10 ns long) on the selected shoulder points and the structures obtained from these trajectories were clustered using the RMSD-based clustering approach to identify the most probable structure representing the deinserted meta-stable state. The value of η and the relative free energy with respect to the global minimum corresponding to this clustering-based most-probable structure are 11.46 Å and 40 kcal mol^−1^, respectively. Using this deinserted structure, we investigated the flipping of partner bases.

At this juncture, it remains unclear whether the β-hairpin insertion precedes or succeeds the flipping of the partner bases and CPD. As the reported *F*(η) was calculated for the experimental crystal structure of the bound Rad4–DNA complex, it is inherently assumed that the partner bases and CPD are flipped out when the BHD3 β-hairpin is deinserted or inserted. It is of interest to estimate the energetics of the β-hairpin insertion when the partner bases and CPD are intra-helical. To this end, additional umbrella sampling simulations were performed on a β-hairpin deinserted intermediate structure of Rad4–DNA complex in which the CPD and the partner bases were flipped inside (Model F). That is, these simulations were carried out with the assumption that the flipping of partner bases succeeds the β-hairpin insertion. The β-hairpin insertion free energy profile obtained for this model is also presented in Figure 5. This free energy profile differs significantly from that obtained from the crystal structure (Model A). For instance, the free energy minimum is shifted to a higher value of η compared to that for the crystal structure. To be specific, η_*min*_ = 3.1 Å for Model A, while η_*min*_ = 10.7 Å for Model F. This implies that the BHD3 β-hairpin is unable to reach the damage site if the CPD and partner bases are intrahelical. Thus, it appears that during the Rad4–DNA encounter the β-hairpin prefers to be in the deinserted state with η_*min*_ = 10.7 Å waiting for the CPD and partner bases to extrude from the DNA duplex. Once they are flipped out, the free energy profile for the β-hairpin insertion is altered such that the energy minimum is shifted to η_*min*_ = 3.1 Å allowing facile insertion of β-hairpin into the lesion site.

The comparison of PMFs between Model A and Model F provides insights into the energetics of β-hairpin insertion and deinsertion from or into the DNA duplex. The basin around the minimum for Model F was broader and more asymmetric than that for Model A, which suggests that the BHD3 β-hairpin is relatively more dynamic near the energy-minimum configuration in Model F than in the other model. In the context of the present discussion, we will regard the states with η = 3.1 Å and η = 10.7 Å as the inserted and deinserted states, respectively. The actual deinsertion energy is the free energy difference between the inserted state (with CPD and partner bases in their intrahelical states) and the deinserted state (with CPD and partner bases in their extrahelical states). However, interpreting the free energy differences from the computed PMFs requires caution. For Model A, the β-hairpin insertion is a downhill process with an energy loss of 41.81 kcal mol^−1^, while for Model F, it is an uphill process with an energy cost of 46.77 kcal mol^−1^. These values cannot be taken directly as measures of the actual deinsertion energy. This is because in Model A, the partner bases and CPD in the deinserted state remained in their extra-helical states, whereas in the actual deinserted state, they are expected to be in their respective intra-helical states. Likewise, in Model F, the CPD and partner bases in the inserted state were found to be in their intra-helical conformations, whereas in reality, they should be in their respective extra-helical states. The free energy differences obtained for the uphill and downhill processes can provide a more meaningful measure of the deinsertion energy, which can be calculated as the difference between the two values. In this case, the absolute value of the deinsertion energy is estimated to be 4.96 kcal mol^−1^.

### Flipping of CPD partner bases

We now proceed to investigate the mechanism and energetics of the flipping of the partner bases (3′-dA and 5′-dA) at the DNA damage site in the different models of the Rad4–DNA complex. As mentioned earlier, in the crystal structure of the Rad4–DNA bound complex, the β-hairpin of BHD3 is inserted into the lesion site leaving the partner bases and the CPD lesion in their respective extrahelical states. However, this static structure proves insufficient to elucidate whether the flipping of the partner bases and CPD precedes or succeeds the β-hairpin insertion. In order to understand the mechanism of base flipping, it is necessary to examine the base flipping dynamics both prior to and post insertion of the β-hairpin of BHD3 into the lesion site of DNA. The challenge is that, in the post-inserted state, as the BHD3 β-hairpin is deeply inserted into the damage site it acts as a major blockage to the entry of the partner bases into the intrahelical state. As a result, the extra-helical to intra-helical transition of the partner bases and CPD is a rare event whose timescales are beyond the reach of the conventional molecular dynamics simulations. The biased MD simulations can be used to forcefully push the partner bases and CPD to their respective intrahelical states. These biased MD simulations use biased potentials to sample high-energy configurations and examine how the BHD3 β-hairpin responds to the forced flipping of partner bases and CPD. However, it is a computationally expensive exercise. On the contrary, in the pre-inserted metastable state in which the BHD3 β-hairpin is about to be inserted into the damage site, the flipping of the partner bases and CPD can be relatively easier than that in the post-inserted state. However, the crystal structure of this pre-inserted state is not available and we need to model this state. The other challenge is that it is unclear whether the partner bases flip in a concerted fashion or in a sequential manner (the flipping of one base is followed by the other). Even in the sequential flipping, whether 3′-dA flips first or 5′-dA flips first is not clear. We need to consider all these possibilities to draw some meaningful conclusion on the mechanism of base flipping in Rad4–DNA complex. Taking into account all these possibilities, we need to investigate the concerted and sequential flipping of partner bases both in the post-inserted and pre-inserted states. Given the limited availability of experimental studies specifically addressing the mechanism of flipping of two neighboring bases in damaged DNA, arriving at a definitive conclusion regarding the preference of sequential flipping over concerted flipping is challenging. Nonetheless, existing research provides supporting evidence that favors the likelihood of sequential flipping as the more predominant mechanism (77,80). Therefore, in this study, we investigate the sequential flipping of bases using umbrella sampling simulation.

To determine the sequence of the flipping events of the partner bases 3′-dA and 5′-dA, four umbrella sampling studies were conducted: (a) 3′-dA flipping right after β-hairpin deinsertion (Model B) using the collective variable δ , (b) 5′-dA flipping on the deinserted Model A having an intrahelical 3′-dA (Model C) using the collective variable γ , (c) 5′-dA flipping right after β-hairpin deinsertion (Model B) using the collective variable γ′ and (d) 3′-dA flipping on deinserted and 5′-dA flipped in structure (Model D) using the collective variable γ′ .

#### 3′-dA flipping before 5′-dA flipping in deinserted state (Model B)

**Figure 6.**
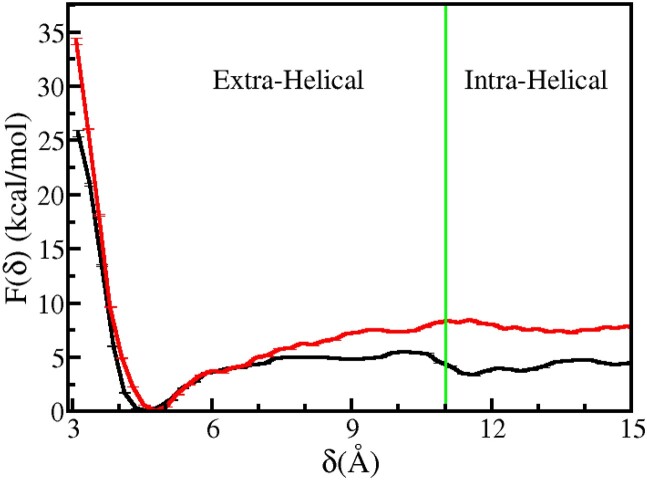
PMF profile associated with the flipping of 3′-dA is shown as a function of δ for Model B (black) and Model D (red).

The calculated free energy profile as a function of δ  is shown for the deinserted state of Rad4–DNA complex (Model B) in Figure 6 (black). A global energy minimum is observed at δ_*min*_ = 4.57 Å, which corresponds to the extra-helical state of 3′-dA in which 3′-dA aromatically stacks with PHE434 residue of Rad4. For δ  < δ_*min*_  the free energy steeply increases due to the steric clashes between 3′-dA and the key residues at the BHD2/BHD3 groove of Rad4. For δ  > δ_*min*_, the interactions of 3′-dA with Rad4 gradually weaken with increasing δ  and *F*(δ ) plateaus around 4 kcal mol^−1^ at higher δ  values ( δ  > 11.5 Å). The observed plateau region (11.5 Å < δ  < 17 Å) corresponds to the intrahelical state of 3′-dA, where it primarily interacts with the neighbouring nucleotides of the DNA.

#### 5′-dA flipping after 3′-dA flipping in deinserted state (Model C)

**Figure 7.**
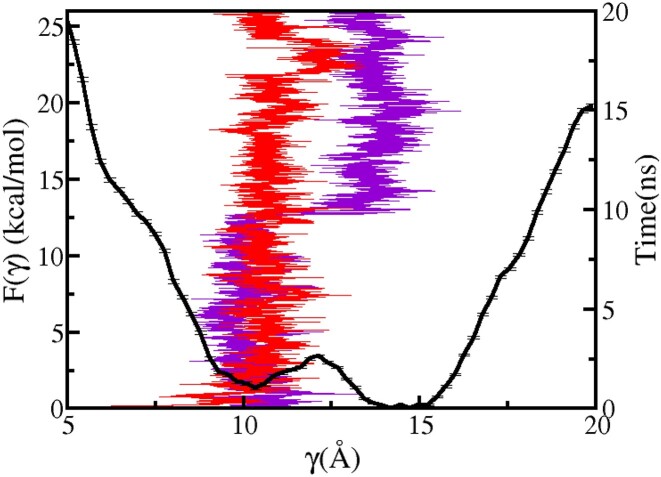
PMF profile associated with the flipping of 5′-dA is shown as a function of γ for Model C. The time series of γ obtained from two unbiased MD runs (red and violet) are also shown.

The calculated free energy profile, *F*(γ ), associated with the flipping of 5′-dA shortly after 3′-dA is flipped into the DNA duplex (Model C) is shown in Figure 7. Throughout this flipping simulation, 3′-dA remained in its intrahelical state. F(γ ) exhibits two energy minima; a global minimum at γ  = 14.2 Å and a second minimum at γ  = 10.4 Å, which is 1.1 kcal mol^−1^ higher in energy than the global minimum. These two minima are separated by an energy barrier, which is located at γ  = 12.1 Å, of 3.19 kcal mol^−1^. In the most-stable global minimum conformation at γ  = 14.2 Å, 5′-dA is in its intrahelical state and it aromatically stacks with the neighbouring base A18_u_. Figure 7 also shows the time series of γ  obtained from 2 independent 20 ns unbiased MD trajectories starting from different initial structures with different γ  values. In one of these unbiased simulations, the system remained in the energy basin around the metastable minimum at γ  = 10.4 Å throughout the trajectory, whereas both the energy minima are sampled by the system during the course of the other simulation. As these barrier-crossing transitions between energy minima are infrequent and rare in unbiased MD simulations, these trajectories cannot be readily used to calculate statistical measures pertaining to these transitions. However, the existence of two energy minima is corroborated by the MD-derived time-series of γ. For γ  >   14.2 Å, the free energy value F(γ ) is seen to increase due to the distortion of DNA around 5′-dA.

#### 5′-dA flipping before 3′-dA flipping in deinserted state (Model B)

**Figure 8.**
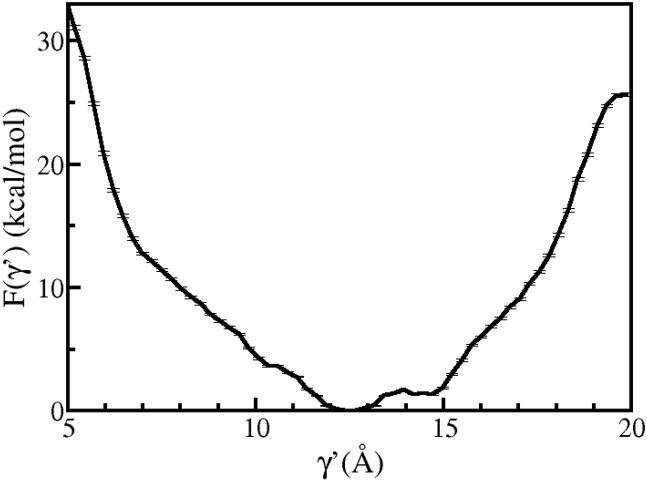
PMF profile associated with the flipping of 5′-dA is shown as a function of γ' for Model B.

Figure 8 shows the free energy profile for the flipping of 5′-dA in Model B calculated using the umbrella sampling method along the γ′ coordinate. A global minimum is observed at 12.57 Å, which is referred to as $\gamma^{\prime }_{min} \,$, where 5′-dA interacts with its neighbouring base A18_*u*_. In the range of 11 Å - 15 Å, the F(γ′ ) value remains more or less constant due to the interaction between 5′-dA and A18_*u*_. However, for γ′ values greater than 15 Å, an increase in F(γ′ ) is observed.

#### 3′-dA flipping after 5′-dA flipping in deinserted state (Model D)

The base flipping free energy profile for Model D shown in Figure 6 (red) exhibits a global minimum at a distance of 4.76 Å, which is referred to as δ_*min*_. At this point, 3′-dA engages in aromatic stacking with PHE434. When δ < δ_*min*_, there is a rapid increase in the value of *F*(δ) due to steric clashes between 3′-dA and PHE434. When δ_*min*_ < δ < 6 Å, a sharp rise in the value of *F*(δ) is observed due to a decrease in the interaction between 3′-dA and PHE434. Beyond δ > 6 Å, the interactions between 3′-dA and PHE434 become minimal. At around δ = 12 Å, the profile becomes completely flat as 3′-dA is entirely flipped inward and interacts with its adjacent nucleotide.

#### Order of flipping of partner bases in deinserted state

A comparison of the free energy profiles for the flipping of 5′-dA nucleotide after (Figure 7) and before (Figure 8) the flipping 3′-dA reveals insight into the most likely order of extrusion of partner bases in Rad4–DNA complex. In both of these free energy profiles, energy minima are observed at γ  values corresponding to the intra-helical region (γ > 9 Å), but the nature of these profiles differ based on the position (intra- or extra-helical) of the 3′-dA nucleotide. Thus, it is evident that 5′-dA base prefers to be intra-helical when the BHD3 β-hairpin is deinserted from the lesion site. The observed differences in the free energy profiles can be attributed to the fact that 5′-dA can favourably stack with 3′-dA in the intra-helical state when its flipping succeeds that of 3′-dA. For γ    ∼ 10 Å, the PMF value in Figure 7 is lower than that in Figure 8 by around 3 kcal mol^−1^. This indicates that it is easier for 5′-dA to flip out when 3′-dA is flipped in. Also, during multiple unbiased and biased simulations, it was observed that out of 3′-dA and 5′-dA, 5′-dA had more liberty to translate/flip and change it’s conformation from being completely extra-helical to intra-helical. This observation is also consistent with the energy profile in Figure 7, here the energy difference between the flipped-in state (minima) and flipped-out state (metastable state around γ  = 10 Å) of 5′-dA is 1.3 kcal mol^−1^. Whereas, the energy gap between these two states of 3′-dA in Figure 6 (black) is ≈ 5.1 kcal mol^−1^. This is due to the fact that 3′-dA is in aromatic stacking with PHE434 which is a comparatively stronger interaction than the non-bonded interactions between 5′-dA and MET376. Thus, by looking at this reaction in a forward direction it can be said that 5′-dA flips first and 3′-dA later, which is inline with previous studies (47,60,79).

#### Flipping of partner bases in inserted state

Thus far, we examined the flipping of the partner bases when the BHD3 β-hairpin was deinserted from the DNA duplex. In order to further comprehend the flipping mechanism of partner bases and the order of BHD3 β-hairpin insertion and base flipping, it is essential to examine the flipping of the partner bases when the BHD3 β-hairpin is fully inserted into the DNA duplex.

**Figure 9.**
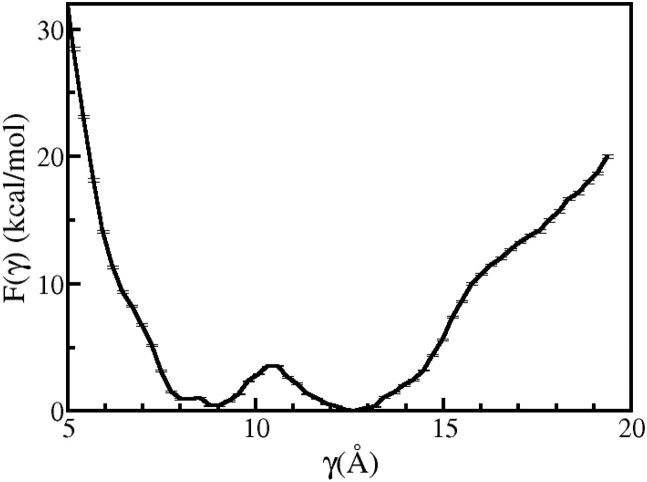
PMF profile associated with the flipping of 5′-dA is shown as a function of γ for Model A.

**Figure 10.**
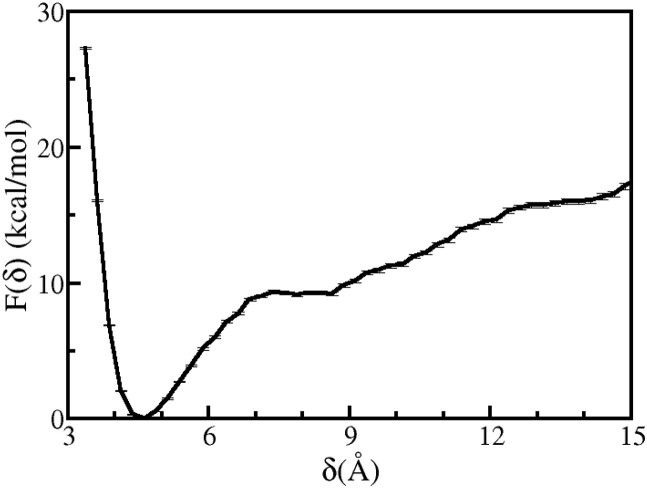
PMF profile associated with the flipping of 3′-dA is shown as a function of δ for Model A.

To this end, umbrella sampling simulations of the flipping of the partner bases 3′-dA and 5′-dA in the bound Rad4–DNA complex with the inserted BHD3 β-hairpin were carried out using γ  and δ  collective variables, respectively (Figure 3C,D). The computed free energy profiles for the flipping of 3′-dA and 5′-dA are shown in Figure 10 and Figure 9, respectively. The global minimum observed at 4.64 Å in Figure 10, which is consistent with the experimental value of 4.75 Å, corresponds to the extra-helical state of the 3′-dA base. The location of this minimum is also consistent with the minimum observed in the corresponding free energy profile for the two previously discussed models (4.57 and 4.76 Å for Model B and Model C, respectively). The energy basin around the minimum is slightly asymmetric and the free energy increases monotonically for δ  > 8.5 Å suggesting that the intra-helical state of 3′-dA is relatively unstable compared to its extra-helical state. This implies that 3′-dA will never reach its intra-helical state when the BHD3 β-hairpin is inserted into the DNA duplex.

A comparison of Figure 10, Figure 6 (black) and Figure 6 (red) reveals that the energy increases with increasing δ  for δ  > 9 Å for the current inserted model, whereas for the deinserted models, the free energy plateaued in this range of δ . The increase in energy in the current model for δ  >9 Å can be attributed to steric clashes between 3′-dA and residues in BHD3 β-hairpin. The free energy profile for the flipping of 5′-dA exhibits two iso-energetic minima (one at γ  = 8.85 Å and the other at γ  = 12.58 Å) separated by a barrier of ∼ 3.2 kcal mol^−1^. Given that the value of γ  calculated from the experimental crystal structure is 7.73 Å, the minimum at 8.85 Å is 1.12 Å away from the experimental value. Thus, it appears that these minima neither correspond to the extra-helical state nor the intra-helical state of 5′-dA. The intra-helical state cannot be reached because of the presence of the BHD3 β-hairpin at the lesion site. The shallow energy basins around these minima suggest that 5′-dA is dynamically more flexible than the 3′-dA base.

To summarize, our findings suggest that CPD and its partner bases must be flipped out to allow for BHD3 β-hairpin insertion during lesion detection by Rad4, that 5′-dA exhibits greater conformational flexibility than 3′-dA, and that the flipping out process occurs sequentially with 5′-dA flipping out before 3′-dA.

### Dissociation of Rad4 from DNA

**Figure 11.**
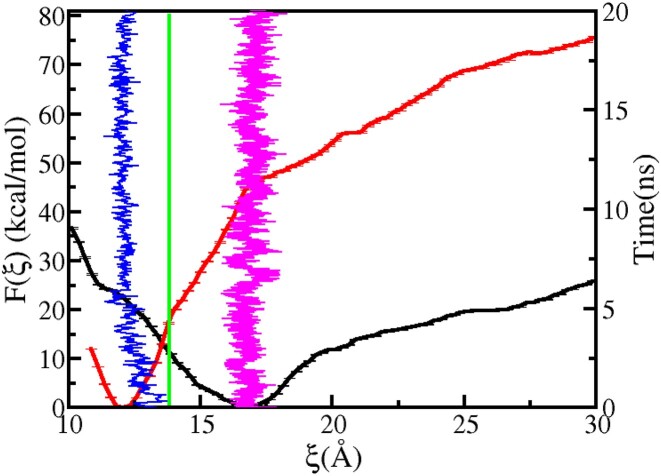
PMF for Rad4–DNA dissociation calculated for the crystal structure (red; Model A) and the encounter complex (black; Model F) in which the partner bases and CPD are intrahelical and the BHD3 β-hairpin is deinserted. The time series of the dissociation CV ξ obtained from unbiased MD runs of Model A (blue) and Model F (magenta) are is also shown.

To investigate the dissociation of Rad4 from the lesion-containing DNA, we first considered a model of Rad4–DNA complex in which both the partner bases and CPD are in their intrahelical states and the BHD3 β-hairpin is deinserted from the lesion site of the DNA (Model F in Figure 4). Figure 11 shows the free energy profile, F(ξ ), for Rad4–DNA dissociation calculated using this model. The global energy minimum of *F*(ξ) occurs at ξ  = 16.9 Å, denoted as ξ_*min*_ hereafter, with an asymmetric basin surrounding it. The increase in *F*(ξ) for ξ < ξ_*min*_ is steep because of steric clashes between the DNA and the BHD2/BHD3 domains. On the other hand, the gradual weakening of favourable interactions between the BHD2/BHD3 domains and DNA causes an increase in *F*(ξ) for ξ > ξ_*min*_. The slope change at around ξ = 19.5 Å suggests that the critical interactions that stabilized the bound complex are almost lost for ξ ≥ 19.5 Å.

The free energy profile, *F*(ξ), was also computed for the experimental crystal structure of the Rad4–DNA complex (Model A in Figure 4) in which the partner bases and CPD are in their respective extrahelical states, and the BHD3 β-hairpin is inserted into the DNA duplex at the lesion site. This profile is also shown in Figure 11. The energy minimum’s location and the nature of the free energy surface differ significantly from those of the former model. The energy minimum is located at ξ  ≈12 Å, which is close to the experimental crystal structure value of ξ  = 13.81 Å and corresponds to the truly associated state of the Rad4–DNA complex. The location of this global minimum more or less coincides with the position of a hump observed at ξ ∼ 11.5 Å in the corresponding PMF for the former model. A comparison of the two free energy profiles reveals that it is less energetically costly to displace Rad4 away from DNA by 3 Å (that is, from the energy minimum) for the former model than for the latter model. The energy cost for ξ − ξ_*min*_ = 3 Å is 11.14 kcal mol^−1^ for the former model and 27.67 kcal mol^−1^ for the latter model. Based on our earlier investigation of Rad4-bound mismatched DNA, this energy cost was estimated to be ∼9 kcal mol^−1^, which is relatively lower than that for the CPD-containing matched DNA (60). This suggests that Rad4 is more likely to remain in an associated conformation for an extended duration with a CPD-containing matched DNA compared to a TTT/TTT mismatch-only DNA.

Based on these results, a plausible mechanism for Rad4–DNA association is proposed. The binding partners initially approach each other and form an intermediate complex with ξ  = 16.9 Å, in which the partner bases and CPD are intrahelical. Subsequently, the flipping of CPD and partner bases occurs, causing alterations in *F*(ξ), such that the energy minimum shifts to ξ  = 12 Å. This shift allows Rad4 to penetrate deeper into the lesion site, forming the final bound complex. The energy difference (*F*(ξ = 11.5 Å) − *F*(ξ_*min*_ = 16.9 Å) between the global minimum and the hump state provides an estimate of the energy cost for flipping out of the CPD and partner bases from the DNA duplex, which is approximately 23.9 kcal mol^−1^. The flipping energy cost for the partner bases and CPD reported in the previous sections sum up to approximately this value.

**Figure 12.**
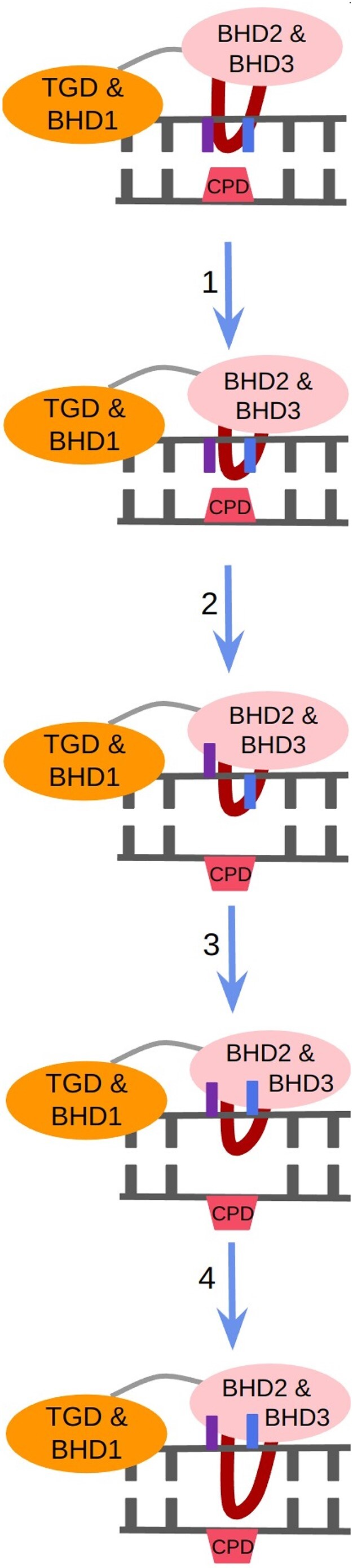
Proposed mechanism for CPD recognition by Rad4. 1. Association of BHD2/3 domains of Rad4 with the DNA; 2. Flipping of 5′-dA (violet); 3. Flipping of 3′-dA (blue); 4. Insertion of BHD3 β-hairpin into the lesion site.

To summarize, a close examination of the energy profiles associated with dissociation of Rad4 from DNA, flipping of partner bases and CPD, as well as the insertion of the BHD3 β-hairpin, yielded two conclusions: firstly, the flipping of 5′-dA occurs prior to that of 3′-dA, and secondly, these flipping events happen before the BHD3 β-hairpin is inserted into the DNA duplex. Figure 12 summarizes the final proposed pathway (or the sequence of events) for CPD recognition by Rad4.

## CONCLUSION

DNA damage can lead to numerous deleterious consequences including cancer and several genetic disorders. The DNA repair proteins recognize and repair DNA damage with high fidelity and safeguard genome integrity. The most prevalent UV-induced DNA lesion known as the cyclobutane pyrimidine dimer (CPD) plays a causal role in skin cancer and skin-related diseases. Rad4/XPC is a key repair protein that senses, interrogates, and verifies the presence of CPD lesions in DNA and then recruits other repair factors to rectify the damage. The intriguing question of how Rad4/XPC locates damaged bases in the genome packaged in a crowded cellular milieu has attracted a great deal of interest among researchers in the field of DNA damage and repair.

The prevailing view of the mode of action of Rad4/XPC in DNA damage recognition suggests among others the three key molecular events: (a) the association of Rad4/XPC with the damaged DNA, (b) the insertion of a lesion-sensing β-hairpin of Rad4/XPC into the damage site and (c) the flipping of a pair of nucleotide bases at the damage site. Given the rugged underlying potential energy landscape of this complex system, these molecular processes turn out to be necessarily slow, making them particularly difficult to investigate using conventional molecular dynamics simulation.

In this study, we have employed an enhanced sampling method in combination with molecular dynamics simulations to investigate the molecular mechanism and energetics of aforementioned molecular events, as well as any potential coupling between them. We initiated our study from the experimental crystal structure of the Rad4–DNA complex and generated eight different intermediate models of this complex, incorporating variations in the positioning of CPD and partner bases (intra- or extra-helical), as well as the presence or absence of BHD3 β-hairpin at the lesion site and Rad4 association with DNA. Given that each of these models represents a potential end state of an intermediate process within the overall NER mechanism, we computed free energy profiles for these intervening processes using appropriate collective variables. Our findings offer significant insights into the sequential order of key events that transpire during the NER process, encompassing the flipping of partner bases, insertion of the BHD3 β-hairpin into the DNA duplex, and Rad4–DNA association.

Our results reveal that it is likely that the flipping of partner bases occurs before the insertion of the β-hairpin during the lesion recognition by Rad4. In other words, the β-hairpin can access the lesion site only when the partner bases are flipped out of the DNA duplex. When the partner bases were forcefully constrained to remain intra-helical during hairpin insertion, the β-hairpin could attain a metastable state located 10.7 Å away from the lesion site. The flipping of the partner bases was found to be a sequential event, with the extrusion of 5′-dA occurring before that of 3′-dA. Furthermore, the conformational flexibility of 5′-dA was found to be higher compared to 3′-dA, with the former exhibiting greater conformational heterogeneity. The reduced conformational flexibility of 3′-dA in the extra-helical state can be attributed to its aromatic stacking interaction with PHE434 of Rad4.

In conclusion, our investigation has provided important insights into the energetics and mechanism of lesion recognition by Rad4/XPC. However, we have only touched the tip of the iceberg, and much more remains to be explored in this area that requires further investigation. For example, multidimensional enhanced sampling methods involving more than one collective variable would be necessary to capture the complex lesion recognition dynamics and to understand the coupling between the aforementioned molecular events. Additionally, a comparative study of Rad4’s binding to different DNA lesions, such as CPD and 6-4PP, as well as mismatches may be needed to gain insights into the similarities and distinctions between the different lesions and mismatch recognition mechanisms by Rad4. Furthermore, the role of co-factors that may modulate Rad4’s function in the NER process can also be explored. We hope that further research in this area will strive to address these open questions and expand the understanding of the complex molecular events underlying the NER process.

## Data Availability

The data described and utilized in this manuscript is available at this URL: https://doi.org/10.5281/zenodo.8128947.
